# A good autistic life: an autistic-led conceptualization of autistic flourishing through autistic women’s-lived experiences

**DOI:** 10.3389/fsoc.2025.1611803

**Published:** 2025-09-24

**Authors:** Åsa Hedlund, Maria Eriksson Wester, Pia Edenvik, Cecilia Ingard, Kajsa Isakson, Lisa Kron Sabel, Danielle Unéus, Therese Lindström, Melissa H. Black, Hanna Bertilsdotter Rosqvist

**Affiliations:** ^1^Faculty of Health and Occupational Studies, Department of Caring Sciences, University of Gävle, Gävle, Sweden; ^2^Independent Researcher, Falun, Sweden; ^3^Department of Social Work, Criminology and Public Health Sciences, University of Gävle, Gävle, Sweden; ^4^Independent Researcher, Stockholm, Sweden; ^5^Disciplinary Domain of Humanities and Social Sciences, Faculty of Arts, Department of Game Design, Uppsala University, Visby, Sweden; ^6^Center of Neurodevelopmental Disorders (KIND), Department of Women’s and Children’s Health, Centre for Psychiatry Research, Karolinska Institutet & Region Stockholm, Stockholm, Sweden; ^7^Department of Community and Clinical Health, School of Allied Health, Human Services & Sport, La Trobe University, Melbourne, VIC, Australia; ^8^Faculty of Arts and Social Sciences, Department of Social and Psychological Studies, Karlstad University, Karlstad, Sweden

**Keywords:** autism, autistic-led research, autoethnography, neurodiverse, women

## Abstract

**Introduction and objective:**

Interest in developing an understanding of “autistic flourishing” is steadily increasing in research and autistic communities. Flourishing is a multidimensional construct explained somewhat by positive emotion, but mostly by good psychological and social functioning. Autistic people process information and stimuli differently from neurotypical people, so it may be reasonable to assume that their definition of flourishing and the factors that influence it may differ from those of neurotypical people. Exploring flourishing from autistic women’s perspectives is essential, as they have been historically overlooked in autism research, despite differing from autistic men in presentation and facing higher mental health risks.

**Methods:**

This autistic-led, partly collective, autoethnographic study was conducted within the context of a broader project exploring the concept of autistic flourishing. Here, we employ a two-phased phenomenological approach, drawing on both autistic and neurotypical frames of analyses. In the first phase, autistic women draw on their lived experiences in a collective autoethnography, including both focus groups and collective writing, to shape the concept of flourishing and its indicators. These insights were further developed by neurotypical authors, who compare to neurotypical experiences and conceptualizations of flourishing.

**Results:**

Two themes and twelve subthemes were identified. The first theme, “Living with a neurodivergent bodymind,” presents how autistic women define and experience flourishing. The second theme, “Strategies for autistic flourishing,” highlights actions autistic women take to achieve or maintain flourishing.

**Discussion and conclusion:**

Through our autistic-led approach drawing on neurodiverse frames of analysis, our work presents a first initial investigation of autistic flourishing among women. Our findings suggest qualitative differences in autistic derived definitions of flourishing and its indicators compared to those of neurotypicals, emphasizing the importance of developing an autistic-driven understanding of flourishing.

## Introduction

There is a growing interest among researchers and the broader community in developing understandings of “autistic flourishing” in contrast to a more common focus on autistic suffering that has dominated research and practice. (c.f. Pellicano and Heyworth, 2023). Given what we know about autism—that autistic people process information and experience stimuli differently (Heasman et al., 2024)— it is reasonable to assume that flourishing among autistic people is defined differently and influenced by other factors compared to flourishing among neurotypical people. In this autistic-led paper embedded in a phenomenological approach to autism, ‘autistic phenomenology’ (Green and Shaughnessy, 2023), we explore autistic flourishing through autistic women’s lived experiences. Drawing on autistic and neurotypical frames of analyses (c.f. Schneid and Raz, 2020), we develop initial conceptualizations of autistic flourishing or a “good autistic life”; what it is, and how it can be achieved. Understanding autistic flourishing presents opportunities to remove barriers to autistic flourishing and to develop support and interventions that enable autistic people to live good lives, according to their own standards. Autistic women, in particular, have highlighted the need for research that prioritizes supporting their well-being (Grove et al., 2023). Since autistic women present differently and are at a higher risk than autistic men of developing mental health problems, we need a gender-conscious understanding of autistic flourishing and gender-sensitive tools to support it (Martini et al., 2022; Lai et al., 2023).

### Gender-conscious understandings of autistic flourishing

Autism was previously thought to be a predominantly male condition. Recent evidence suggests that many autistic girls and women have been under- or misdiagnosed because autistic traits can manifest differently in girls and women compared to boys and men, and experiences of autism can differ across genders (e.g., Alaghband-Rad et al., 2023; Kiep and Spek, 2017). Autistic women may struggle more than autistic men because higher pressure to conform to gendered expectations (Yau et al., 2023). Masking or camouflaging – known to be associated with adverse mental health outcomes - though reported in both autistic men and women, appears to be particularly extensive among autistic women (Remnélius et al., 2024; Hull et al., 2020; Rynkiewicz et al., 2019). In addition, autistic girls and women face a higher prevalence of depression, anxiety, and other mental health problems than their male counterparts (Martini et al., 2022). Exploring autistic flourishing through a gender-conscious lens can help to address the challenges and barriers faced by autistic girls and women, ultimately supporting them to live a “good autistic life.”

### Autistic suffering: traditional approaches

Since autism was first identified within a Western medical context, research has largely been grounded in deficit-oriented approaches, rooted in the notion that being autistic is equivalent to suffering (Chapman and Carel, 2022; APA, 2013). As a result, research and practice have focused on remediating “deficits” in order to reduce this perceived “suffering.” This large body of research tends to emphasize limitations defined in contrast to neurotypical ideals, focusing on risks and negative outcomes such as morbidity, and mortality. (e.g., Shtayermman and Fletcher, 2022). Autistic people do indeed report a range of challenging experiences, including cognitive or sensory overload (Lewis and Stevens, 2023), camouflaging or masking of autistic characteristics (Pearson and Rose, 2021), and burnout (Raymaker et al., 2020). Such experiences can ultimately erode quality of life, mental health, and overall wellbeing. Autistic women are particularly vulnerable to mental health issues, such as depression and anxiety disorders (Oswald et al., 2016; So et al., 2021). However, more recent literature suggests that these challenges arise from the interaction between intrinsic aspects of autism and barriers and exclusion within a predominantly neurotypical world, with such external factors potentially amplifying their severity and impact (Grove et al., 2023; Pearson and Rose, 2021).

### Shifting from autistic suffering to autistic flourishing

In contrast to deficit-based narratives centred on suffering, the concept of flourishing focuses on thriving in ways meaningful to the person and the pursuit of a “good life.” Embedded in positive psychology, this perspective shifts attention to actively enhancing and promoting a good life. Flourishing shares similarities to other concepts such as happiness, well-being, life satisfaction, resilience, and growth, but goes beyond mental states or singular metrics of so-called “success” or thriving, to an all-encompassing holistic perspective in which “all aspects of a person’s life are good” (Vander Weele, 2017, pg. 2). This is in line with the WHO (1948) definition of health as “a state of complete physical, mental and social well-being and not merely the absence of disease or infirmity.” In literature on neurotypical flourishing, the construct has been conceptualized in diverse ways. One conceptual analysis, drawing on the extant literature, proposed the following definition: “living a personally meaningful and engaged life. It is a multidimensional construct explained somewhat by positive emotion, but mostly by good psychological and social functioning” (Agenor et al., 2017, pg. 920). Similar to its overarching definition, its defining attributes vary across the literature. While meaning, positive relationships, engagement, competence, positive emotion and self-esteem are commonly shared across the literature, other domains have also been proposed (c.f. Vander Weele, 2017), underscoring that flourishing remains a flexible and evolving concept.

In the context of autism, a flourishing perspective presents a crucial role in promoting a genuinely “good life” for autistic people. There are indications that autistic flourishing or what “a good autistic life” may look like, may differ from that of the neurotypical population, as autistic people experience and interact with the world differently than neurotypical populations (Heasman et al., 2024). Ross et al. (2023) examined caregiver-reported flourishing items and found that caregivers of autistic children perceived their children as experiencing lower levels of flourishing compared to caregivers of neurotypical children. However, evidence of measurement bias in the items raises concerns about the suitability of this tool, suggesting that traditional flourishing measures may fail to capture autistic children’s actual experiences/true flourishing. Chapman and Carel (2022) further argue that autistic and other ways of being that differ from neurotypical flourishing have been excluded from the flourishing discourse, proposing that flourishing should not be viewed through only a neurotypical lens. Pellicano and Heyworth (2023) further propose a need to move away from a solely neurotypical lens, suggesting the following five building blocks as a foundation for autistic flourishing:

1. Shifting from a more limited perspective on autistic flourishing, focusing solely on conventional physical and mental health, to a broader understanding of well-being (p. 420)
2. Moving from an externally defined to an internally defined perspective on flourishing (p. 421)
3. Moving from “the big to the small” – i.e., “a range of everyday concerns that build up to be the weft of ordinary life” (p. 422)
4. Shifting from the individual to the “Individual-in-Context,” including “the need to move from an individual understanding of flourishing to a contextual one” (p. 423)
5. Moving from (neurotypical) researcher-led to autistic-led, stressing the importance of autistic people as “being partners in the research” (p. 423)

In contrast to the fifth suggestion, however, in this paper we instead seek to push slightly toward a modified approach, what Jackson-Perry and Bertilsdotter Rosqvist (2024) have referred to as an inverted inclusive research approach in neurodiversity studies, where neurotypical researchers –.

“willing to recognize and reflect upon their knowledge as being situated, are those *being* included, not those *doing* the including.” Informed by this approach, our paper adopts a neurodiverse frame of analysis – combining autistic and neurotypical frames of analyses – where all neurotypes are reflexive about their own situated knowledge and impacts of their frame of analyses. However, it is also autistic-led as autistic people lead the research, and neurotypical researchers are being included (a reversal of typical patterns). Through our neurodiverse frame of analysis, we intend to contribute to a gender-conscious understanding of autistic flourishing.

## Methods

### Design

The current study draws on an autistic collective autoethnography informed by an autistic phenomenological approach. Autistic members of the author group draw on our embodied lived experiences to inform our conceptualization of flourishing, which aligns with both feminist theory (Ahmed, 2017) and the notion of autistic-led theories (Murray, 2018), where theoretical models and understanding are drawn from the personal lived experience, and at least partly internal observations. The whole author group consists of autistic (HB and ÅH), and neurotypical (MHB and TL) researchers within the broader project (described below), members of the autistic co-creation group of the project (CI, KI and DU) and autistic members of the reference group of the project – consisting of health professionals - (MEW, LKS and PE.).

### Context

This study is a part of a wider Swedish research project on autistic flourishing and ways to develop and support a ‘good autistic life’ (funded by the Swedish Research Council). The overall aims of this wider project are to: (1) explore the concept of flourishing from an autistic perspective, (2) examine how public services can incorporate and support autistic-flourishing approaches, and (3) co-create a consensus to guide the design and delivery of supports and interventions that align with autistic flourishing. Approaching the first aim of the broader project, we set out to conduct interviews with autistic people on the topic of flourishing. During the process of developing the interview guide which was led by the autistic researchers (HB and ÅH), assisted by members of the co-creation group and autistic members of the reference group, we came to realize we needed to do something more than just thinking about possible interview questions, how to measure autistic flourishing, and interpretations of the interview questions. The autistic members of the author group felt the need to experience the questions themselves and, throughout this process, develop the interview guide, coming to a formulation of a pilot within the project. The process of experiencing and interpreting the questions led us to develop our first initial conceptualization of autistic flourishing, which is the focus of this study.

### Positionality

We are a neuro-mixed group of people, assigned female at birth living in a high-income Western country who share the experience of identifying as women and being subjected to gendered expectations and norms. Autistic authors include late-identified autistic people as well as AuDHD people. All of us are interested in neurodiversity research, and several of us work as researchers in the field. Some of us have a social sciences background, others come from psychological or medical research. Many of us bring a clinical health care/health promotion perspective stemming from training in various health professions (e.g., nursing, medicine, psychology, occupational therapy). Others have a background in social work/sociology, in education, or art/design. We approach this work from a bio-psycho-social perspective, viewing human functioning as arising from interactions between the individual’s strengths and challenges and the facilitating and hindering factors in the environment. We all bring our situated knowledge when reading, analyzing, and interpreting the study results. Important to note, though, is that the interpretive prerogative, decision-making process, and conceptualization of autistic flourishing remained grounded in an autistic perspective throughout, with the neurotypical frame of analysis functioning more as a complement, a contrast.

### Data production

Data was produced through three digital focus group interviews via Zoom in January and February 2025, as well as ongoing reflection by autistic authors during the introductory stage of analysis. The interviews were led by ÅH (the interviewer) and each lasted around 60 min. ÅH, HB, MEW, and LKS participated in the first interview; ÅH, CI, KI, and P in the second; and ÅH, HB, and DU in the third. Questions were based on a semi-structured interview guide, covering topics such as how autistic flourishing is experienced, what it looks like, in which situations it occurs, what individuals can do to promote their own flourishing, and what external support contributes to it. The goal was not to ask every question exactly as written; rather, the interview guide served as a tool for the interviewer to initiate the interview and keep the conversation within the topic throughout. We then allowed the discussion to take its natural course. The interviewer acted to moderate the discussion, but also at times participated as a respondent, representing their dual role as a researcher and autistic woman. To accommodate everyone’s cognitive energy, we decided to limit the interview to 60 min: 5 min for introduction, presentation, and information about the interview process, 40 min for the interview itself, and 15 min for evaluating the questions and interview method (explored elsewhere). The interviews were audio- and video-recorded. Another accommodation we did was to allow for post-interview processing. Following the interviews, all participants were encouraged to share any additional thoughts on autistic flourishing that had emerged after the interview and upon reflection. Reflections could be shared via discussions with the interviewer, in written form, or by adding additional thoughts continuously during data analysis and co-writing the paper. This meant that there was no clear end of the data production stage, but it rather continued alongside the process of data analysis.

### Data analysis: introductory and continuation analysis

Analysis was performed in two steps. In the *introductory stage of analysis*, the autistic members of the group of authors conducted a qualitative inductive thematic analysis (Braun and Clarke, 2021) through an autistic frame of analysis. The interviews were transcribed verbatim using the generative AI program Amberscript. Following transcription, ÅH reviewed the transcriptions while listening to and watching the recordings to correct any errors and add notes on body language. ÅH and HB conducted the preliminary analysis together. We identified and highlighted data units, i.e., parts of the text that were considered relevant for the research topic. Those were then sorted into groups depending on their content, i.e., similar content was grouped and given initial themes. Data on both a semantic and latent level were analyzed, considering not only the explicit meaning of our words but also, when data was considered deep enough, the underlying social context and deeper implications. As we wrote the running text under each initial theme, a clearer picture of the themes’ content emerged, leading to a revision of their names. We could also see that the themes were grouped into two larger categories addressing similar aspects, leading to the formation of two main themes. The themes and their content were subsequently reviewed by the remaining autistic authors, who had direct access to the raw data to ensure that their own autoethnographic descriptions aligned with the thematic interpretations. This process allowed for a reflexive engagement with the data, where authors critically reflected on their experiences in relation to emerging themes. Their input helped to validate and refine the themes, ensuring that the analysis remained grounded in the lived experiences represented in the data. Following this collaborative review, the themes were further revised, with additional reflexive thoughts incorporated to enhance the depth and rigor of the analysis.

We have chosen to refer to the autoethnographic voices in the text with a collective “One of us.” This is a way of stressing the text as written in a collective space, the collective “I” as “One of us.” “One of us” is also used as an expression of a “joint action” which feminist researchers Francis and Hey (2009, p 231) have stressed as a “core to feminist action over the years” but in particular within academia” where joint action counter-narrate the position as “individual experts.” The phrase also has deep roots in the disability rights movement, where it has been used as a marker of solidarity and self-representation, reclaiming belonging from outsider definitions and resisting exclusionary narratives. The use of a collective I, is a way to counter-narrate the image of “the sole,” individualized neurodivergent, and rather stress the presence of a neurodivergent togetherness but also to protect ourselves against structural violence and position ourselves– as a neurological minority (stigmatized) selves.

In the *continuation stage of analyses,* the neurotypical authors (MHB and TL) reviewed the themes derived from the autoethnographic approach through their neurotypical frame of reference. The neurotypical authors first examined and discussed their personal framings and experiences of flourishing, drawing on extant literature that has described flourishing from neurotypical perspectives. They then reviewed the themes derived from the autoethnographic approach and reflected on how they position and interpret the findings through their neurotypical lens. There was an emphasis on comparing and contrasting the results of the autistic frame of analysis to the neurotypical perspective, seeking to identify both similarities and differences in the conceptualization of flourishing. These reflections were shared with the autistic researchers within the project (HB and ÅH) and discussed. Points of divergence and convergence in the conceptualization of flourishing through autistic and neurotypical frames of analysis are discussed in the discussion section.

### A note on researcher standpoints and the impact of different frames of analyses

As this project is autistic-led and seeks to define a neurodiverse frame of analysis -combining autistic and neurotypical frames of analyses- the neurotypical authors were not involved in the first stages of the research process, data production, or introductory analysis. However, throughout the process of writing we came to realize the possibilities of combining autistic insider with neurotypical outsider perspectives, however in a new way: acknowledging the need to develop another frame of analyses than the one commonly used in autism research – where research are neurotypical-led (with decision making in research by neurotypical researchers) and the neurotypical gaze (McDermott, 2022) or neurotypical frame of analyses is commonly invisible and taken for granted (similar to other normative gazes, such as a heteronormative, white or male gaze). Given the often inherent but covert power structures that may make the neurotypical frame of analyses dominant, our approach ensured that the decision-making process remained firmly grounded in an autistic-informed perspective. Rather, the neurotypical authors provide interpretations and insights through their neurotypical frame of analysis, complementing the autistic frame of analysis. Our neurodiverse approach within this work is believed to contribute to new insights beyond those that could be obtained from work generated within a single frame of reference alone.

Following recent developments in the field of phenomenological inquiries into autism (Murray et al., 2023; Green and Shaughnessy, 2023), the autistic researchers’ frame of analysis was based on a combination of different kinds of autistic theorizing:

1. Embodied experiences of functioning and interacting with the world in a way that does not align with that of the neuro-majority.
2. Largely detailed-oriented (bottom-up) cognitive style impacting ways of thinking.
3. Full members in the researched group or setting, informed by previous and ongoing discussions in autistic communities.
4. Embodied theorizing where theoretical models and understanding are drawn from a combination of the personal lived experience, and at least partly internal observations, and in interaction within the autistic group of authors.

In contrast, the neurotypical researchers’ frame of analysis can be described as:

1. Embodied experiences of functioning and interacting with the world in a way that aligns with that of the neuro-majority.
2. Largely holistic-oriented (top-down) cognitive style impacting ways of thinking.

This project is autistic-led because its focus is autistic flourishing, and it is essential that autistic authors hold first interpretive rights over their own experiences. At the same time, our aim is to work as a neurodiverse team, recognising that a full picture emerges only when autistic and neurotypical perspectives meet in genuine dialogue. Neurotypical frames of analysis — often invisible and taken for granted in autism research (McDermott, 2022) — are made explicit here, not to diminish their value, but to situate them as one perspective among many. Our approach is therefore not about excluding or ranking perspectives, but about ensuring that historically marginalised autistic perspectives are centred in this work while also valuing the complementary insights that neurotypical frames bring. By consciously navigating differences in cognitive styles, processing pace, and focus (c.f. Bertilsdotter Rosqvist et al., 2023; Pearson et al., 2024), we aim to develop a shared, reflexive neurodiverse frame of analysis that neither defaults to neurotypical norms nor erases them, but uses the tension between perspectives to deepen our understanding of diverse ways of functioning. Our differences in cognitive styles (largely bottom-up versus largely top-down) means we consciously need to develop ways in which our differing cognitive styles can “meet up” and support each other, but differences in processing pace and what we “see” in the data can also pose challenges which needs to be translated (c.f. Bertilsdotter Rosqvist et al., 2023) and navigated in relation to internal ableism and years of masking (c.f. Pearson et al., 2024).

## Results

We arrived at two main themes and twelve subthemes (see Table 1). The first main theme, “Living with a neurodivergent bodymind,” captures how the autistic authors describe and experience autistic flourishing. This includes both how we conceptualize it and its attributes. The second main theme, “Strategies for autistic flourishing,” focuses on what the autistic authors actively do to achieve or maintain the experience of autistic flourishing. Each subtheme is supported by quotes from us (the autistic authors) and is presented in italics. We also acknowledge that not all of the experiences described are exclusive to autistic people, though some of them are likely to be.

**Table 1:** Overview of the main themes and sub-themes.

Themes	
*Living with a neurodivergent bodymind (attributes of autistic flourishing)*	*Strategies for autistic flourishing*
Energetic vs. grounded and balanced as a whole	Allowing oneself to feel bad / listening to emotions and bodily signals
Sensory stimulation / seeking sensory pleasures	Directing one’s focus
Needing stimulation / escape from boredom (sensory and cognitive underload)	Reducing sensory and cognitive overload
The importance of understanding one’s own way of functioning/Understanding oneself from a non-pathological perspective	Using another person to sort thoughts and understand experiences/seeking validation of experiences
	Being in genuine contact with another person / engaging in interest-based interaction
	Adapting social situations to ones ‘own need
	Choosing one’s social environments
	Planning

### Living with a neurodivergent bodymind (attributes of autistic flourishing)

#### Energetic vs. grounded and balanced as a whole

Describing what autistic flourishing means proved challenging, not because we lacked experiences to draw from, but because the available words often felt too broad or too imprecise to capture the specific ways we live and feel it. By grounding our discussion in lived experience, we were able to reveal forms of flourishing that are inseparable from sensory realities and patterns of perception often invisible in broader cultural narratives.

*For me, emotions have always been complex because they almost always came along with sensory dysregulation—something I didn’t realize I was experiencing. It was only when I started seeing myself through an autistic perspective that I could begin to differentiate between emotions and sensory overload.*

*In addition to a different experience and interpretation of perception/interoception, I also seem to 'have' what is called alexithymia. I constantly have to intellectually piece together different elements into a pattern in order to understand what my emotions 'represent'.*

Autistic flourishing was described as having two aspects: on the one hand, a high-energy state that was primarily experienced in the mind, and on the other hand, a low-energy state that was more about balance and being present in the body. We described the high-energy aspect as experiencing pleasure, high energy, high pulse, joy, being in flow (“immersed in an activity”), in hyperfocus, feeling that something is exciting, being in the right rhythm, feeling creative, being “high” on interest-driven motivation, feeling stimulated (as opposed to bored/underloaded/under-stimulated), “vibrant energy,” and a sense that everything is amazing. Being in these states was described as mentally enjoyable, but at the same time, we experienced that it could put stress on the body. Many of us found it challenging to find a balance between mind and body. One of us described it like this:

*On one hand, I am energetic and full of energy, kind of like right now. I’m way too high on this interest-driven state. It’s like, “Oh God, this is exciting! Oh God!” And in my head, I feel really great. But my body is quite stressed. I feel it all over my body (thump thump thump). And at the same time, when I’m focused on my body, I actually become non-speaking, and I enter a kind of hyperfocus that is very sensory and physical. That really happens when I’m in an intimate relationship with someone—whether it’s sexual, or when I’m lying next to the person I’m holding, my partner. It’s a very cocoon-like feeling where all my senses become focused on just a few sensations … I feel completely harmonious. But then my head is gone (small laugh), like, it just doesn’t work. For me, those two states don’t fit together.*

The more low-energy side of autistic flourishing was described as a sense of being grounded, present, still, balanced, calm, safe, open, fearless, proud, soft, harmonious, content, capable, and at ease. We described it as being within a window of tolerance or a sweet spot, moving with lighter steps, having a less cluttered mind, feeling a strong sense of self that comes from within (not influenced by how we think others perceive us), being aware of the world around us while still feeling grounded, feeling dissolved and at one with everything, being free from “bad” or uncomfortable emotions, not feeling self-conscious (e.g., How am I being perceived? Is there something wrong with my body?), and experiencing the mind running (or flowing, associating) freely. We also touched on more bodily descriptions—fully inhabiting one’s body, being immersed in physical sensory experiences, feeling connected to the whole body (as one of us put it, being “with myself”), having a sense of ease in the stomach, and experiencing the body functioning perfectly in motion. The low-energy side of autistic flourishing feels sensory in nature, and we become non-speaking—that is, we fully engage in the moment through wordless observation and sensory experience.

Flourishing was not necessarily a static state but rather fluctuated over time. Many of us talked about how it is usually difficult to balance attention between the external world and inward focus, but that there seems to be a kind of balance in the low-energy side of autistic flourishing:

*… it is a combination of being very present and clear, both with oneself and with the surroundings in some way, while at the same time being in a very relaxed state. So, a combination of some kind of presence and relaxation … and specifically, being able to hold both the external and internal at the same time.*

Several of us felt that grief and other difficult emotions resulting from negative life events do not necessarily exclude the experience of autistic flourishing. Autistic flourishing also seems to be something that can be experienced multiple times a day, alternating with moments of intense discomfort. As one of us described:

*I experience things moment by moment, and it can take time to look back and remember what I just felt. It’s a bit like I don’t 'carry' the emotional state from one moment into the next. If you were to ask me here and now how I’m feeling, and I were to answer based on this exact moment, you would likely get a different answer than if you had asked me a minute ago. Because then, I might have been in a completely different experience.*

### Sensory stimulation/seeking sensory pleasures

We described various forms of sensory stimulation as contributing to autistic flourishing. The most discussed aspect was visual stimulation, such as being in and experiencing the aesthetics of nature or appealing environments, e.g., the way light falls over or streams through trees, and when light, sound, and color return to nature in spring. One of us described how intensely the craving for visual stimulation can make up the entire day’s experience of meaning.

*If I haven’t been out in nature for a few days, my longing, my need can become so intense that it feels like I’m going to break down if I don’t get access to it. Like life is being taken away from me if I don’t get to experience aspects of nature. If I can then go outside to watch the sunset or other beautiful aspects of nature, it feels like I immediately come into balance. I feel happy, satisfied, and restored. I feel alive, and the experience gives me meaning.*

We also talked about the pleasure of noticing beautiful details in our surroundings by focusing on patterns and textures, for example, a twisted branch in nature or an appealing pattern on a wall in a room. We found this kind of quiet absorption to be deeply enjoyable, which meant we preferred environments rich in detail (as opposed to bare or “boring” spaces) but without being visually overwhelming. One of us emphasized that every detail needed to be beautiful in order for the experience to be pleasurable:

*But just, uh, that … that every part must be beautiful. Then the whole thing becomes beautiful. Otherwise, if there's something that isn’t beautiful, it disrupts the entire … the entire overall impression.*

Other sensory pleasures mentioned included sound, taste, touch, and scent. Music, in particular, was described as both pleasurable and helpful for managing difficult emotions (such as anger or sadness) and for enhancing positive emotions and other inner experiences (such as strength, nostalgia, or calm). Some of us also saw it as a biological necessity:

*… if I haven’t listened to music for a while, it’s like my brain craves rhythm and melodies. And when it gets to that, I feel satisfied. It’s almost as if it’s a biological need in some way—for the brain to receive these patterns that it pieces together, or whatever it is.*

Several of us described how we create playlists based on how they feel in the body:

*It’s more like sensory-seeking stimming. So listening to music that feels good in the body. That’s actually how I make my playlists—it’s purely based on how it physically feels in my body.*

Eating foods we found delicious, such as pastries and chips, offered moments of sensory delight that supported our sense of flourishing. Touch in specific areas could be a pathway to autistic flourishing as well. One of us described how having someone touch her back made her feel completely relaxed, both mentally and physically, and how—due to often having a tense nervous system—she felt she needed daily massages. Another described how she loved working with garden soil, both for how it feels and how it smells:

*… it’s the sensation itself—playing with the smell and the feeling of the soil in my hands. And then I can fully immerse myself in it, and I feel everything flowing out, the difficult emotions disappear, because I go into hyperfocus with the soil.*

### Needing stimulation/escape from boredom (sensory and cognitive underload)

To avoid sensory and cognitive underload (under-stimulation and boredom), we engage in many different and complex activities.

*Diving deep into subjects and thinking in a complex way feels like a basic need for me. When I don’t have access to that, I feel more down and lose my sense of vitality.*

Stimulation was described as important for alleviating impatience, restlessness, and low energy, but also as the experience of being able to “use oneself,” which was an important attribute of flourishing. Examples of this included engaging in creative hobbies (such as crafting, baking, or cooking), having stimulating jobs, studying, or engaging in physical activities like running or climbing. The key was to have multiple activities, not just one. Several of us described work (employment) as one of the most enjoyable things in life when we work with something that aligns with our way of functioning and our interests. We want to work a lot, using our creativity and problem-solving skills. One of us (who is a health care worker) described the satisfaction of taking on and succeeding in complex work tasks:

*I manage to do something at work that feels advanced. Amazing! Now I’ve really solved a problem well. I’ve helped someone, relieved their pain. Someone feels safe with me before surgery. Someone who is seriously ill gets something from me. It gives so much life satisfaction to be part of something and do a task well, one that took a long journey to get to the position where I can actually perform it.*

Performance at work was described as a byproduct of doing something enjoyable, rather than a goal in itself. We would rather go through “effort and sweat” than be passive. One of us described how her flourishing depends on stimulation and how she constantly moves along a scale between zero stimulation and very high stimulation—with her placement on this scale being the main determinant of her well-being (unlike how many others describe it, in terms of loneliness vs. social connection or health vs. illness):

*It’s like stimulation is the only thing that matters in some way—whether it’s physical or mental. And the more stimulation I get, the better I feel.*

Another one of us described how stimulation through genuine connection is crucial for experiencing well-being in social interactions:

*If I don’t feel that genuine connection in every moment of an interaction, then the whole interaction can instead feel dead and meaningless to me.*

Several of us described the joy of giving and the feeling of empathy for others as part of our flourishing. Because of this, many of us have chosen human service professions. One person described the joy of helping people who are struggling, while another spoke about being a support to people in general. Many of us described a strong desire to alleviate the suffering of others. One of us put it this way:

*I have come to see myself as a modern witch—someone who wants to help but refuses to be limited by rigid societal rules. Like a catalyst, I want to help people flourish and become their best, most wonderful selves.*

### The importance of understanding one’s own way of functioning/understanding oneself from a non-pathological perspective

Self-knowledge was described as essential for experiencing autistic flourishing, but not just in the sense of “knowing how I function.” It was also about understanding oneself from a non-pathological perspective. Seeing ourselves as whole and with self-love makes a huge difference in our well-being. This means not blaming ourselves after being in a situation where we did not fit in, but instead recognizing that it was the situation that did not fit our way of functioning. As one of us said:

*It was only after understanding that I am autistic that I could begin to feel love toward myself and receive it from others. I’ve been able to set aside all the dysfunctional assumptions I had about myself and experience a sense of being grounded. I’ve started to understand what goes against my neurology/information processing, which has given me a greater sense of self-agency. Because of this, I can choose my environments more intentionally, and I can also explain my behaviors more easily to others (which, as a minority, is still a task that falls on me). And I can be kinder to myself when an environment doesn’t align with how my brain works. Having this knowledge has been the key to taking control over my own well-being.*

Another person described:

*Something else that has been important for my own autistic flourishing is processing all the ableism I had internalized. My relationship with myself has improved so much as I’ve been more accurately mirrored by others, and as I’ve been able to define and understand myself beyond the deficit perspective.*

The ability to comprehend, on a profound level, the way you function and also understand in what ways others may have a different way of functioning is an important prerequisite for successful social interaction. This internal awareness also makes it possible to explain your way of functioning, helping others to understand you.

*When I “came out” as an autistic person at work, I got a lot of loving remarks and much support and understanding. Although the word autistic was not enough, I had to specify what it signified in my case, since many people had a hard time accepting it, as I didn’t fit their preconceptions of autism. Still, my life has truly improved, with less frustration and conflicts. I have also learned to communicate more clearly and ask more questions when I don’t understand something. I no longer have to pretend I am someone I am not.*

### Strategies for autistic flourishing

#### Allowing oneself to feel bad/listening to emotions and bodily signals

We described how we allowed ourselves to listen to our body’s signals to enhance the experience of autistic flourishing. Several of us seem to consciously use stimming to take control over what experiences to enhance and what to reduce. And one of us explained how she allows herself to feel bad and accepts it, which paradoxically makes her feel better. Another person described something similar—not “fighting” or avoiding the negative but relating to it in a more positive way, as one of us put it:

*… I try to see anxiety as a friend rather than an enemy—like, it wants to tell me something, not to be mean, but because, in some way, it wants what’s best for me.*

### Directing one’s focus

We talked about focus—how it could be directed, used, and experienced to achieve autistic flourishing. Several of us described creating an inner world to generate a sense of calm and joy. This was described as a kind of daydreaming, inward attention, or role-playing. One of us described escaping into inner spaces, creating an environment that she feels she needs in the moment:

*If I could choose completely freely, where would I want to be right now? And then I create that space. It brings me calm.*

Another person described how she sometimes pretends to be another character, while still being herself, as a way to make the day more enjoyable or cozy:

*… Maybe I’m a, uh … university student, and, uh, when I sit down, I imagine myself—as a student—setting up my desk, making it cozy for myself, and just … And I keep reinforcing that throughout the day, and it’s something that just makes everything much easier and more pleasant. Cozier.*

Another one of us described how she creates things to look forward to, as this makes her feel good in the present and results in a better day. These did not have to be big things—it could be something as simple as reading something interesting and then eating something delicious.

Some of us did not engage in inner imagination in that way but instead described the experience of being in hyperfocus, which was seen as “wonderful” and enjoyable, even though switching focus could be difficult and energy-draining. One of us described it like this:

*When I enter hyperfocus, I go higher up, and I become even more. And then I become very … Afterward, I drop down into a kind of, uh, 'crash, ' as I call it.*

Another person described hyperfocus less as an internal state of feeling and more as deep immersion into a specific subject. It was about the interest-based desire and drive to understand something (c.f. Murray et al., 2005), such as people and how they function physically and mentally. One of us, who is both autistic and ADHD, described how hyperfocus is linked to both autism and ADHD, and how the two can enhance each other (c.f. Dwyer et al., 2024). However, she felt that her hyperfocus was more tied to autism, as it involved absorbing all available material and seeing all the connections clearly:

*The difference between my ADHD hyperfocus and my autism hyperfocus is that the first one makes me jump into the unknown, while the second one is more about reading and learning something interesting. But both are pleasurable. The autism hyperfocus is more about depth, analyzing layer after layer, absorbing all knowledge, and then more. It’s pleasurable, long-term, and comes in waves. My ADHD hyperfocus works quickly with logistics, organizing all time-related layers, checking them off. It’s more about doing—sometimes physically, sometimes mentally, solving problems and moving forward fast. It’s active, sensorimotor, dancing, stimming—an intense tempo in the moment.*

### Reducing sensory and cognitive overload

We talked about the measures we take to avoid sensory and cognitive overload, as ways of assisting ourselves in achieving flourishing. One of us described how, after experiencing burnout, she had learned to work more sustainably—reducing her effective working hours and shifting from “over-efficient” to a more “normal” work pace. We emphasized the importance of a flexible workplace that allows us to structure the day according to our own energy patterns. Another one of us described how she stims a lot to stay grounded, sometimes together with her child, who she described as creative in coming up with fun new stims. We also discussed the need to be alone (alone time) and how it serves as a way to recharge—being able to focus on our own needs without having to accommodate others. One of us shared that she reduced her working hours to have one day a week to herself. When the interviewer asked how she identifies her needs, she responded that it has become easier after a period of trial and error, observing how her body and brain react to different activities:

*… it felt like my brain was thanking me when I went out for a walk.*

She also described learning when to turn off her phone and avoid screens. For example, she turns off her phone before starting an activity she wants to focus on, as fragmented attention disrupts the mental rest she experiences when she can fully focus on one thing, enter flow, and be present.

After social situations that do not suit us (e.g., under-stimulating environments), we could feel tired or over-thinking, which some of us described as a kind of meltdown. Listening to podcasts was mentioned as an effective way to interrupt rumination and to recover after social situations. Others described needing sleep and waiting until the next day for the overthinking or exhaustion to subside.

*Sleep itself is important—for many of us. Before the overthinking takes over, before everything gets messy. And structure, lists, and order.*

Many of us described being in water as calming – water as a regulating tool, either because of the sensation of floating/lightness or the warmth of hot showers. Some of us emphasized the importance of external feedback from a trusted person —for example, to check how we came across in a social situation—so that we could let go of inaccurate negative self-perceptions. One of us described repeating mantras out loud to silence her inner critical voice, which she likened to “a crow on my shoulder, pecking at me.” Exercise or Physical activity, such as yoga or running, was described as an important strategy for recovery.

*Sometimes, I go out for a run, and then I start thinking—'Oh, what did I say? What did I do? Was that okay? Maybe I should’ve said it differently,' and then I start running faster, and then my brain stops thinking (small laughter from the group). I have to push myself.*

Several of us also preferred to combine exercise with spoken expression—such as saying mantras, shouting, or using rhythmic speech—when alone. Other calming strategies mentioned were: staring into space and being without social input to gradually regain energy, reading books, meditation, handicrafts, or watching television series. Some of us made a difference between the different uses of watching television series: “engaging” watching (watching television series the person like, find either joyful or interesting) or “calming” watching (watching television series not necessarily to the person’s liking or strong interest, but mildly interesting and helpful in breaking thought loops and allowing the brain to rest). The second category includes series that may create a sense of calm—moderately engaging, of average quality, and with few sensory stimuli. Such series could make it easier to mentally switch off, recharge, and gradually wind down and relax. For example, one of us noted:

*I usually prefer rewatching something familiar or choosing something new that aligns with my usual interests or themes I enjoy.*

Some of us acted as our own therapists, using a firm but kind inner voice to guide ourselves back to balance. Others used shock-based strategies, such as cold-water immersion (winter bathing), to regulate the nervous system, lying on an acupressure mat. The goal of these strategies—besides stopping rumination and regaining energy—was to return to balance, feel grounded, and redirect focus to the present moment. One of us expressed that worry and overthinking are incompatible with a sense of vitality—and that it’s simply not worth losing the joy of life over a social situation. This mindset helped her let go of overanalyzing:

*I have no desire to dwell on it. I just have to say ‘fuck them’ and move on. Forward.*

Avoiding working in the evening was also described as important, as it overstimulates the brain and makes it harder to wind down. We also discussed games as a tool for focus and relaxation, preferring stimulating but not overly cognitively demanding mobile games, such as Sudoku or Tetris, which can help both focus and relaxation, as well as interrupt intense hyperfocus. However, for some of us, games became an obstacle to relaxation, as we got stuck in them and had difficulty stopping, even when we knew we needed to sleep.

### Using another person to sort thoughts and understand experiences/seeking validation of experiences

Several of us described how we benefited from discussing with other people to sort and understand our thoughts and to validate them, which helped us prevent or let go of overthinking. However, this required a trusted relationship. One of us described how she can call her mother-in-law at any time to talk about different social situations and get help in sorting through and understanding them. Her mother-in-law does this by sharing her own thoughts on the situation and asking questions. She described it like this:

*But actually, nowadays, I mostly just need to talk through things and feel that someone else receives it and validates my experience in it. And, uh, I find that this helps because otherwise … I mean, I get help putting the pieces together into a whole so that it doesn’t become separate traumas.*

Several others described using journaling in a similar way. The journal functioned as a non-judgmental counterpart, and the act of writing itself helped us sort through, reflect on, and understand our experiences. Clarifying communication with oneself could also happen through inner dialogue, without writing.

### Being in genuine contact with another person/engaging in interest-based interaction

We described how being in authentic contact with others and having interest-based interactions were essential for autistic flourishing. We talked about how social interactions should either be “real” connections (which are easiest to create in safe relationships) or that we should be alone. “Real connection” was described as: Sharing the same energy—both people being engaged and interested in the moment together, feeling comfortable together in silence, associating and developing something together, moving effortlessly in and out of intensity and calm, being in a shared flow and feeling fulfilled by it (physical presence makes this easier, while digital communication makes it more difficult), feeling a calm pulse, love, shared rhythm, kindness, humor, simplicity, and presence, having space to talk about personal interests with someone who is genuinely interested, and maintaining a pace that works for both people. Several of us described how it was important that others did not focus on our facial expressions or body language but instead just listened to what we were saying. One of us described it like this:

*Being in a situation or relational context where the focus isn’t on my expressions, but instead on what we are doing together. And maybe it’s that feeling—that both or all of us are focused on what’s being discussed, or what we’re engaged in, or even just what each of us is doing. But this is a really important part of a truly close relationship for me—that there aren’t any unnecessary interpretations (makes a sound as if shuddering from discomfort).*

One of us described how she has a reflex to constantly self-monitor herself in social situations and how it takes a long time to feel comfortable with new people. To be fully present in social situations, she needs one of three things: a clear task to focus on, an interesting conversation topic, or a certain level of intoxication. These are the only times she can be close to someone without simultaneously watching herself from an external, critical perspective. She described how her sense of social well-being depends on environments where she can interact without constantly self-monitoring. In those spaces, she can be fully present, experience genuine connection, and engage socially without analyzing herself in real-time. Many of us described the importance of having a few, but safe, deep, close relationships—relationships that are loyal, authentic, kind, and where we are appreciated for who we truly are. In these relationships, we can dive straight into a conversation—sometimes without even saying hello—just jumping into something that feels genuinely meaningful. These relationships could be with family, friends, colleagues, or even clients/patients/service users. One of us described it like this:

*Now I have a colleague I work with daily. We spend a lot of time together but move in and out of intense social contact, unfiltered with each other, sharing values. It feels safe, restorative, and enriching. I also get a lot of social well-being from my clinical work as a psychologist. It might sound strange—of course, the relationships with clients are asymmetrical in many ways, I carry the responsibility, and the focus isn’t on me. But for me, it’s a relaxed, interesting, caring, clear, and genuine social environment. It’s closeness in a different way. In those moments, I feel deeply connected to myself. And I feel hopeful about humanity and my place in it—something I rarely feel in the ‘ordinary’ world. It’s probably because the social structure is both structured and free, and because my ability to see patterns, notice details, and hold multiple things in my mind at once is truly useful there. There’s no superficial small talk—everything being said matters, and we engage in what genuinely interests me. It’s like I go into relational/social and intellectual hyperfocus. I understand myself and grow. Maybe it also helps that many of the people I meet in my work are neurodivergent—but it’s not about that, it’s more about the form of social interaction.*

Many of us described how we experience social well-being through parallel play (c.f. Pritchard-Rowe et al., 2024)—that is, being physically together with someone while doing things separately, without needing to interact using spoken language.

### Adapting social situations to one’s own needs

Several of us described different strategies we used to adapt social situations so that they better meet our own needs and foster our ability to flourish. One of the strategies was to shift our focus to make it easier to navigate social situations that do not naturally align with our way of functioning. One of us explained that she uses her interest in people to observe others in the situation, instead of focusing solely on interaction. This way, she becomes engaged in observing, which helps her feel more comfortable. Another strategy was to retreat into an inner space where there is an “imaginary friend” to have a dialogue with about the people and the social situation. One of us described it like this:

*It creates a space, a distance that allows for reflection—just enough to regulate myself, become aware of how I am affected and why. It prevents me from being consumed by discomfort, stiff emptiness, and self-criticism. It helps me integrate my inner and outer worlds. I observe the others. And I accept that I won’t be my best self in these situations—and that’s okay.*

Observing others was described as a way to make social situations more engaging. Another way to make interactions more interesting was to ask others about a topic of personal interest, steering the conversation toward something engaging. If the social situation was a work meeting, it could be framed as just another paid task—something to complete without overanalyzing or questioning, and then move on. One of us described how she sees social communication as a skill to learn, like playing an instrument or even like a game. Viewing it this way made social situations feel more enjoyable. Another aspect of this is strategies to make social interaction more attuned to one’s own pace of cognitive processing. For example, one of us explained that social situations felt more comfortable when she allowed herself to take the time she needed to think and speak, as this made her feel more authentic. This also illustrates strategies to shift to a non-pathological, self-loving perspective. Many of us described shifting focus from “feeling inadequate” to being more forgiving toward ourselves in social situations. We reframed it, not as something being wrong with us, but simply as the situation not fitting our way of functioning:

*… not being so self-blaming … Because it wasn’t my situation … But I did my best.*

### Choosing one’s social environments

We discussed how important it is to be able to choose our social environments. Many of us try to seek out social situations with people we feel comfortable with and avoid those that do not suit us. New relationships were described as more energy-consuming, so we often prefer to spend time with people we already know and who know us well—in other words, “safe” relationships. One of us described how crucial it is to have her boundaries respected:

*So there’s never that feeling, and I’ve experienced that it almost becomes—I wouldn’t call it violence, but something close to that. When I feel tired, I signal it, but the person won’t accept it and says, ‘Oh, we can talk a little longer’, or ‘Are you already tired?’ There are so many things that make it harder to end the interaction. But when I’m with an old friend, it’s just—‘I’m tired’. ‘Okay’. ‘Bye’.*

One of us described how the social environments she enjoys are either simple or stimulating, which she defined as clear, direct, informative, fast-thinking, and focused on fun and interesting topics. Another one of us described that she wants to be in inclusive and loving environments—where she feels understood, liked, and respected. One of us emphasized that being autistic does not automatically mean she functions best with other autistic people. Instead, it depends on the other person’s personality traits, such as whether they communicate directly, so she might just as easily connect well with a neurotypical person. We talked about how we prefer social settings where we are taken seriously, even when we ask unusual or unexpected questions. In the following, three of us explore (with a mixture of serious interest and amusement) conversations about asking ‘odd questions’:

*Interviewer: I tend to ask a lot of questions, but never the usual ones. I never know about people’s pets, grandkids, home renovations, and that kind of thing. Instead, I ask deep questions or just something completely random. Whatever comes to mind.*

*One of us1: Like, 'What kind of toothbrush do you have?' What would that be?*

*Interviewer: Yes, or whatever I’m currently interested in. It could be anything.*

*One of us2: What a wonderful question, I need to think about it. What kind of toothbrush would I be? Hahaha!*

*Interviewer: Exactly! Or, I find it interesting to ask when people were born or how tall they are, for example. And I can go straight into a seemingly random topic if I’m interested in it. If I’m in a setting where people are open, they usually find it amusing. They think it’s a bit funny, which is nice—it’s not always a bad thing.*

*One of us2: Yeah, but I struggle with being seen as ‘a little funny.’ That feels very specific. I don’t know if I want to be ‘a little funny’—I want to be taken seriously. For example, if I ask, ‘I have a red toothbrush, what do you think about that?’ I don’t want the response to be, ‘Haha, how funny!’ That’s not what I meant. I was asking, ‘What kind of toothbrush do you have?’*

We also talked about how communication and social interaction need to have a focus, rather than just being about spending time together aimlessly. For example, two of us reflect upon social activities that feel more comfortable:

*One of us: A few friends and I went to the water treatment plant when they had an open house and a tour. Not because we’re really interested in water treatment plants, but … it just feels easier when there’s something to do.*

*Interviewer: There’s always something to talk about, at least.*

*One of us: Exactly! It’s like—‘we are here to learn about this’ instead of ‘we are here to socialize.’*

Many of us prefer small groups because we function best that way. However, if we do find ourselves in larger groups, one of us described how she turns most of the people into “background characters” rather than focusing on them directly, which makes the situation more manageable. Others said they actually enjoy large groups because they can take “social breaks” and feel less pressure when the focus is not on them all the time. One of us emphasized how important it is to live alone so she can choose when to engage socially:

*… I don’t want people too close to me either. I don’t want someone living in my home, because then I wouldn’t be able to retreat. I want to socialize, and then be able to go home. (small laugh).*

### Planning

Many of us described planning our daily lives as essential. As one of us put it:

*… I plan my week very meticulously. I love planning, and I love following my plan. It makes things quite inflexible, but I’m very satisfied with that.*

Planning was described as stress-reducing and ensuring that necessary activities for well-being—such as taking walks or completing important tasks—actually get done. Different methods were used to establish and maintain plans, such as various types of lists and digital apps with programmed reminders. We not only planned specific activities but also set goals for those activities. One of us described how she used an app that reminded her of tasks. She sometimes felt resistant at first, for example, when prompted to take a walk. But once she was outside, she realized how much she actually needed it:

*And then I have my app. The app with the little bird says, ‘Now you need to go for a walk.’ And I just think, oh god, how boring. But once I’m out walking, I think, oh my god, this is amazing!*

One of us, who is both autistic and ADHD, described how ADHD medication had, for the first time in her adult life, helped her build routines:

*I am so grateful for ADHD medication—it has, for the first time in my adult life, allowed me to build some routines. Stimulant medication has made it clear to me that I actually have a strong need for plans and routines that I can follow. I do, however, feel more unsettled when they are disrupted—but overall, they have significantly improved my baseline mood.*

The planning also had an important function in ensuring that we did not forget to do basic things. To eat, sleep, and exercise regularly and in the proper amount is important for all beings. However, some of us emphasized the importance of this, as it may not come naturally to autistic people, due to different sensory experiences of hunger, and different natural circadian rhythm than the general society. Some of us stressed the benefit of regular exercise to maintain a peaceful mind. Planning for these basic needs was described as an important part of daily life, including regular breaks with rest or exercise and extra snacks to prevent fatigue.

## Discussion

### Contributing to a definition of autistic flourishing

Through our neurodiverse frame of analysis, we have begun to shape our conceptualization of autistic flourishing. Working with a collective autoethnography, we focused specifically on women’s (our) lived experience to collectively explore our understandings of an autistic good life and autistic flourishing. Autistic flourishing, from our exploration, could perhaps be summarized as a bodymind, internal, everyday experience - with balance, authenticity, and energy being core attributes. Many of the descriptions used by us to capture autistic flourishing could be compared to what is described as mindfulness—being present in the moment, experiencing the present through the senses without judgment, focusing on one thing at a time, and taking in a (detail-oriented) reality as it comes to us.

These initial descriptions of autistic flourishing both overlap with and contrast with prior neurotypical conceptualizations. Flourishing for the neurotypical authors, drawing on lived experience and the extant neurotypical literature, revolves around happiness, wellbeing, and personally meaningful fulfillment in areas of life, such as in relationships and productive and personal occupations. In comparing the autistic and neurotypical frames of reference, we see similarities; for example, experiences of well-being, such as feelings of happiness, are also a central aspect of flourishing in the extant literature (Vander Weele, 2017). However, the autistic conceptualizations of flourishing appear more detail-oriented and internally driven - flowing across mental and physical spaces, with a clear link to inner experiences and balance of intellectual and sensory stimulation. This can be contrasted to some social and emotional metrics underpinning traditional conceptualization of flourishing, where relatively more weight seems to be placed on concurrent perceptions of doing well, over time, across valued areas of life. Following this, flourishing is a highly individualized experience. Where the extant literature places emphasis on relationships and occupations (such as work or community participation) as pathways to flourishing, autistic flourishing was rather described as driven by having one’s needs for space, communication, and processing time met, with a particular emphasis on intellectual and sensory stimulation.

Among the challenges we faced in seeking to develop our conceptualizations of autistic flourishing was the demarcation of what qualifies as flourishing. Indeed, this is an issue also faced in the extant literature, with flourishing having several definitions and attributes (Agenor et al., 2017). It is likely that difficulties defining the boundaries of flourishing stem, at least in part, from its inherently all-encompassing nature. We note that there were risks that the concept became too vague and identified conceptual overlaps in our discussions with concepts such as wellbeing and autistic joy. These conceptual overlaps lead to an important conceptual discussion – where neurotypical authors framed these concepts (e.g., feelings of well-being or contentment) as distinct and temporary, viewing them as a component or outcome of flourishing, while autistic authors viewed them as indistinguishable from flourishing itself. These findings point to the importance of our neurodiverse frame of analyses; to combine our differing interpretations.

During the interviews, it became clear that a challenge for autistic authors is the impact of epistemic injustice for autistic theorizing (Fricker, 2007; Chapman and Carel, 2022). Due to a lack of hermeneutic and interpretative resources, autistic authors experienced a lack of autistic language for describing autistic experiences, and that language therefore limits us when we try to explore our experiences. Indeed, it is possible that the limitations of language also influence our internal understanding of ourselves. Some expressed frustration during the interview that it becomes “banal,” “flat,” “shallow,” or that it is difficult to convey the comprehensive response that exists inside. Descriptions of, for example, sensory experiences often fall flat when they have to take shape in established language. We simply lack words to describe our experiences. Established language at hand may not allow for the speed/changeability, richness of detail, nuance, and/or context-dependence needed to capture our internal and external states. Following Chapman and Carel (2022), hermeneutic injustice can contribute to the neurotypical world missing essential knowledge that autistic people have inside but have not formulated together.

For (some of) us, spoken language is also a sensory experience that needs to be created freely, beyond linguistic rules and conventions, where the importance of authentic alignment with what is intended requires precision, repetition, and processing. It becomes important to understand that the taken-for-granted categories within language do not necessarily align with autistic ways of perceiving, expressing, and being in the world. Many of us describe in the interview various forms of receptivity and active behaviors in relation to both the internal and external. Perhaps we experience a greater presence (on a sensory, non-speaking level) of internal processes that neurotypical people remain more unaware of? It is interesting to consider what this means for the limitations of spoken language and the understanding of ourselves.

It is important to note that “high energy flourishing” (e.g., being in hyperfocus) could also have negative consequences, such as exhaustion, which is why recovery (and possibly a “low energy flourishing”) is needed afterward. Autistic flourishing thus also comes with a need to work with both forms of high and low energy, which must be taken into account by those supporting autistic women toward a flourishing life.

### A gender-conscious understanding of autistic flourishing

Our study was embedded in autistic women’s perspective of flourishing, seeking to, in part, balance the historically neurotypical and male-centric focus of autism research. Authenticity and self-awareness were cornerstones to our autistic flourishing. Authenticity can be particularly difficult for autistic people who frequently come into conflict with gender expectations, especially as these expectations are often more prescriptive and demanding for women than for men (Alaghband-Rad et al., 2023).

Much of this self-awareness came first from an autism diagnosis. As girls and women are historically under- and misdiagnosed, this lack of diagnosis may present a barrier to flourishing. The result showed that viewing oneself from a non-pathological perspective was important for autistic flourishing. This can be difficult if one is not even aware that one is autistic. Kentrou et al. (2024) showed that women were even more likely than men to report perceived misdiagnoses of personality disorders, anxiety disorders, and mood disorders. This emphasizes the importance of girls having the opportunity to discover their autistic functioning as early as possible.

One of the central findings of this study is the role of sensory stimulation in our flourishing. Autistic women, like their male counterparts, often seek sensory stimulation (MacLennan et al., 2022). However, societal expectations may place additional pressure on women to “fit in” and suppress these needs (Charlton et al., 2021). As a result, it is crucial to create environments that not only acknowledge but also actively accommodate autistic women’s sensory preferences, allowing them to express their sensory needs without fear of judgment. Providing such support is not just beneficial for their immediate comfort but is also fundamental to promoting both short-term and long-term autistic flourishing. Furthermore, in this study, we found that engaging in personal interests is essential for our sense of flourishing. This supports previous findings by Yau et al. (2023), who also emphasized the positive impact of interests on well-being. However, Yau et al. (2023) also noted that autistic women often struggle to engage in their interests due to a lack of like-minded peers and the societal norms that women are expected to conform to.

As seen in the present study, we struggle to articulate our inner experiences: to name our experiences with an autistic language. This can, as mentioned before, be understood in light of the fact that language is largely shaped by neurotypical conceptualizations, making it difficult for many autistic people to identify, express, and communicate their feelings in a way that feels authentic. For autistic women, this challenge can become even more complex, as they are also influenced by gendered norms around, for example, emotional expression (Yau et al., 2023). As a result, autistic women may be doubly deprived of an autistic language—both due to neurotypical standards and gendered expectations—which can hinder their flourishing.

### Moving forward

Our neurodiverse frame of analysis illustrates the importance of developing autistic-led conceptualizations of autistic flourishing. Autistic-derived definitions of flourishing and the pathways to flourishing seemed to differ from the conceptualizations of the neurotypical authors and those presented in the extant literature. Aligning with neurodiversity approaches to autism, there is a need to move beyond solely neurotypical conceptualizations of flourishing to instead respecting and acknowledging differences in what may entail a “good autistic life”: both in terms of everyday moments of flourishing, as well as short- and long-term pathways to flourishing. Following autistic theorist Joanne Limburg (2022), we can “try and find a language for all kinds of moments that make up human existence,” where “the accumulation of moments, however trivial they were in themselves, can determine the course of a life.” (Limburg, 2022, p 23).

In seeking to capture flourishing for autistic people, we did note some similarities between autistic and neurotypical people. Indeed, experiences of “flow,” “pleasure,” and “joy” are *shared* experiences. However, partly due to the impact of epistemic injustice, the meanings and internal experiences of these words and behaviors might differ between neurotypes. It may therefore be necessary to explore new autistic languages and to understand how intensely something is experienced, and how life-defining or essential an experience or behavior may be.

Following our analyses, we see a need for two fundamental shifts. Firstly, a shift away from deficit-oriented approaches to flourishing-oriented approaches to autism. Secondly, a shift away from neurotypical conceptualizations of flourishing to an autistic-led one. Regarding the first point, research and practice in autism have remained firmly embedded in deficit-oriented approaches that focus on autistic suffering – here, we advocate for a shift toward autistic flourishing, focusing on opportunities to support autistic people to live a good autistic life. However, shifting from a deficit-oriented approach to a flourishing-focused approach is only half the task. Our study suggests that there may be fundamental differences in how flourishing is conceptualized, created, and experienced between autistic and neurotypical people. Efforts may be needed to find a shared understanding of what pathways (e.g., prerequisites, strategies, and priorities) will actually promote flourishing for the individual concerned.

### Concluding reflections

This work presents a first initial investigation of autistic flourishing among women, embedded in an autistic-led approach and drawing on neurodiverse frames of analysis. Through our neurodiverse lens, we have taken our first steps toward a shared understanding of autistic flourishing. The neurodiverse approach presents opportunities to move beyond insights beyond those that might be captured through one frame of analysis alone.

We note key challenges in conceptualizing flourishing, notably limitations of language to capture autistic experiences, and the blurry boundaries of the all-encompassing concept of flourishing. We will attempt to tackle these challenges and refine our understanding of autistic flourishing in our future work. This has important implications for research and practice moving forward, where it should not be assumed that the concept means the same to all people, captures the same experiences, or is underpinned by the same factors.

During the data collection, we noticed in ourselves that available concepts do not always align with our cognitive style, and that language can feel limiting, unable to provide the nuanced, accurate, and detailed descriptions we desire of autistic flourishing. At the same time, language has an enormous power to shape people’s perception of reality. From our reflections on a neurodiverse frame of analyses we see both challenges and possibilities of “meeting up” in different ways of cognitive processing: autistic frame of analyses (largely dominated by details over wholes, using bottom-up processing) in contrast to a neurotypical frame of analyses (largely dominated by emphasize of wholes over details, using top-down processing). Inclusion of neurotypical researchers in the continuation analyses stage was a way to give priority to an autistic frame of analysis. In this paper, autistic-led not only means that decision-making and research design were led by autistic researchers, but also that the analytical process in itself centered autistic cognitive processing and decentered neurotypical cognitive processing. By this, we want to stress challenges associated with the unreflective use of (largely top-down) language and categorizations in autism research, as this may risk misinterpretations or predominantly neurotypical (outsider) interpretations of autistic flourishing.

Even though we identify as women, we want to emphasize that binary categorization of gender is problematic in the context of autism. Neurotypical expectations of gender may not reflect autistic people’s actual experiences of themselves. Questioning gender identity, breaking away from the cis-norm and the binary view of gender is common among autistic people (Mittertreiner et al., 2024). Neurotypical expectations of gender may risk reproducing and reinforcing limiting stereotypes of autistic people and what it means to flourish, through which new knowledge is filtered. Diagnostic criteria and assessment tools have been created within a patriarchal structure, understanding autism both with a cis-man as the norm and from a neurotypical perspective. The expected image of autism is therefore limited to behaviors that clearly deviate from gendered expectations of neurotypicality. However, making “autism in girls/women” visible should not be done by creating a new category/subtype of “female autism,.” We find a perception of “female/male autistic” functioning problematic. Combinations of gender-based expectations and the prevailing conceptions of autistic functioning are clearly restrictive. It risks limiting representations and the complexity of autistic people’s reality. Instead, it is about recognizing a broad spectrum of autistic functioning, combined with the consequences of gender structural injustices, which risks rendering particular experiences of female or nonbinary people invisible or misinterpreted. Gender-based expectations (as well as other power structures) themselves contribute to negative outcomes, such as mental health issues, for both neurotypical and autistic people. To enable autistic flourishing, it seems autistic people of all genders need to be aware of our autistic way of functioning, as well as have an intersectional understanding of the structures/expectations we live within and how these affect our interpretations of ourselves and the world around us.

## Data Availability

The raw data supporting the conclusions of this article will be made available by the authors, without undue reservation.
